# TyroFill–Titanium Implant Constructs for the Coordinated Repair of Rabbit Mandible and Tooth Defects

**DOI:** 10.3390/bioengineering10111277

**Published:** 2023-11-02

**Authors:** Weibo Zhang, Joachim Kohn, Pamela C. Yelick

**Affiliations:** 1 Department of Orthodontics, Division of Craniofacial and Molecular Genetics, Tufts University School of Dental Medicine, Boston, MA 02111, USA; 2 New Jersey Center for Biomaterials, Rutgers University, Piscataway, NJ 08854, USA

**Keywords:** dental pulp stem cells, alveolar bone regeneration, tyrosine-derived polycarbonate scaffolds, bone remodeling, titanium dental implant

## Abstract

Currently used methods to repair craniomaxillofacial (CMF) bone and tooth defects require a multi-staged surgical approach for bone repair followed by dental implant placement. Our previously published results demonstrated significant bioengineered bone formation using human dental pulp stem cell (hDPSC)-seeded tyrosine-derived polycarbonate scaffolds (E1001(1K)-bTCP). Here, we improved upon this approach using a modified TyroFill (E1001(1K)/dicalcium phosphate dihydrate (DCPD)) scaffold-supported titanium dental implant model for simultaneous bone–dental implant repair. TyroFill scaffolds containing an embedded titanium implant, with (*n* = 3 each time point) or without (*n* = 2 each time point) seeded hDPCs and Human Umbilical Vein Endothelial Cells (HUVECs), were cultured in vitro. Each implant was then implanted into a 10 mm full-thickness critical-sized defect prepared on a rabbit mandibulee. After 1 and 3 months, replicate constructs were harvested and analyzed using Micro-CT histological and IHC analyses. Our results showed significant new bone formation surrounding the titanium implants in cell-seeded TyroFill constructs. This study indicates the potential utility of hDPSC/HUVEC-seeded TyroFill scaffolds for coordinated CMF bone–dental implant repair.

## 1. Introduction

Craniomaxillofacial (CMF) defects remain a significant health concern where trauma, cancer, and birth defects can cause significant physical and psychological impacts [1,2,3]. Critical-sized CMF defects cannot heal on their own due to their limited regenerative potential. Therefore, the repair of CMF defects requires highly specialized surgical interventions to restore proper form and function. Currently used CMF repair therapies require a lengthy, multi-staged surgical approach for bone augmentation, followed by surgical dental implant placement. One of the main challenges for these therapies is to ensure the successful regeneration of sufficient bone to provide reliable support for the dental implant. Autologous bone grafting remains the gold standard for CMF defect repair therapies based on its superior properties with respect to immune response and biocompatibility [4]. Clinical applications for bone repair by xenograft and allograft therapies are limited by concerns regarding potential immune rejection, exposure to viral contaminants, inadequate bone regeneration, donor site morbidity, and unpredictable bone graft survival [5]. To date, tissue engineering approaches using autologous bone-forming cells are considered to be the most promising therapies for effective bone defect repair [6,7].

Another unique consideration for repairing the CMF complex is the fact that craniofacial bones have a distinct embryological origin as compared to the axial and appendicular skeleton [8]. Craniofacial bones, including the jaw bones, arise from neurectoderm-derived neural crest cells, while other bones are of mesodermal origin [9]. CMF bones, including the tooth-supporting alveolar bone that resists resorption in response to the strong forces of mastication, differ from axial and appendicular bones with respect to their response to mechanical and homeostatic stimuli [10]. Moreover, many CMF bones undergo intramembranous as opposed to endochondral ossification [11]. Craniofacial bone grafts show improved volumetric maintenance and survival when used in CMF repair [12], although bone marrow-derived mesenchymal stem cells (MSCs) derived from mesoderm [13,14] are most commonly used for craniofacial bone regeneration. This is due to the relative scarcity of CMF bones and the likelihood of disfigurement by harvesting procedures. Human dental pulp stem cells (hDPSCs), which can be harvested from extracted deciduous, wisdom, and other teeth, have the distinct capability to not only form tooth related tissues, but also mineralized tissues exhibiting characteristics of alveolar bone [15,16,17]. Advantages for using hDPSCs for CMF tissue repair include the fact that they share the same embryonic tissue origin—they are both derived from the neural crest—and hDPSCs can easily be harvested from teeth that would otherwise be discarded. In addition, hDPSCs have the demonstrated capacity to regenerate a variety of craniofacial tissues, including composite jawbone and dental tissues.

Another important consideration for tissue-engineered regenerative therapies is the type of scaffold to select for large defect repair. Synthetic polymer/ceramic scaffolds are some of the most commonly used scaffolds for bone regeneration due to the fact that they are biomimetic and osteoinductive [18]. Tyrosine-derived polycarbonate (TyrPCs) scaffolds, recently developed by the Kohn laboratory, have been extensively characterized for applications in bone regeneration [19]. To date, TyrPC family-derived porous scaffolds fabricated from 90 mol% DTE, 10 mol% DT, and 1 mol% PEG (MW = 1 kDa), abbreviated as E1001(1k), have been shown to support robust bone regeneration in calvarial and long bone defect repair models, particularly when the E1001(1k) scaffolds contained calcium phosphate [20,21,22]. Moreover, more soluble forms of calcium phosphates for bone scaffold fabrication have been widely investigated for bone engineering strategies [23]. Based on these promising properties, we investigated the utility of hDPSC-seeded E1001(1k)-bTCP scaffolds for alveolar bone regeneration in a small animal rat ramus defect repair model [24]. Based on our promising results in the rat, we next demonstrated that tyrosine-derived polycarbonate E1001(1K)-bTCP scaffolds seeded with hDPSCs and Human Umbilical Vein Endothelial Cells (HUVECs) supported the formation of abundant alveolar jawbone regeneration in a critical-sized rabbit mandible defect repair model [25]. In the rabbit study, we observed active bone remodeling by both osteoblasts and osteoclasts present on newly formed bone surfaces in all implants, and in particular, the implants seeded with human DPSC/HUVECs. Together, these studies demonstrated the utility of hDPSCs-seeded E1001(1K)-bTCP scaffolds for superior bioengineered alveolar bone regeneration. These studies also show that the rabbit mandible and tooth defect repair model serves as a robust, mid-sized animal model for human craniomaxillofacial regenerative therapies. Limitations to the rabbit model include the fact that only one 10 mm full-thickness defect can be made in each rabbit, limiting the sample size. Rabbit studies allow for in vivo validation prior to conducting clinically relevant studies in a large animal minipig craniofacial defect repair model.

We next wanted to determine whether further modification of E1001(1k)-bTCP scaffolds could improve their bone regeneration properties enough to support a dental implant. We, therefore, replaced bTCP with a coating of dicalcium phosphate dihydrate (DCPD, CaHPO_4_·2H_2_O), also known as brushite, which is known to facilitate improved bone metabolism [26]. The Kohn laboratory fabricated E1001(1k)-DCPD scaffolds (now referred to as TyroFill scaffolds) and demonstrated that TyroFill supports bone formation when transplanted into non-load-bearing rabbit calvarial defects [27]. We next characterized the in vitro behavior of hDPSCs-seeded TyroFill scaffolds, demonstrating comparable biocompatibility and osteoconductivity of hDPSCs as compared to hDPSCs-seeded E1001(1K)-bTCP scaffolds [28].

The long-term goal of our research is to develop more effective therapies for the coordinated regeneration and functional repair of CMF defects that reduce costs and patient recovery times. Therefore, in this study, we tested a new model for coordinated, simultaneous jaw bone–dental implant placement and regeneration, using TyroFill scaffolds supporting a titanium dental implant, in a critical-sized rabbit mandible defect repair model.

## 2. Materials and Methods

### 2.1. Preparation of TyroFill (E1001(1k)-DCPD) Scaffolds

The fabrication of E1001(1k)-DCPD (here referred to as TyroFill) scaffolds consists of the following steps: porous E1001(1k) scaffolds were prepared by porogen leaching [29] followed by the formation of a DCPD coating within the pores of the scaffold [28].

Step 1: Briefly, 2 g of E1001(1k) polymer was dissolved in 1.4 mL of deionized (DI) water and 8.6 mL of 1,4-dioxane overnight. An amount of 18 g of NaCl (particle size 212–425 μm) was placed into a Teflon mold. The polymer solution was then slowly poured over the NaCl and allowed to diffuse throughout the salt bed for 1 h. The Teflon mold was covered during that time. After 1 h, the Teflon mold was frozen rapidly in liquid nitrogen and then freeze-dried for 48 h. Disk-shaped scaffolds (10 mm diameter, 6 mm height) were punched out from the Teflon mold. These disks were kept in DI water overnight to leach out the salt crystals. The leached scaffolds were dried in a lyophilizer (Stella Freeze Dryer, Millrock Technology, Kingstson, NY, USA) for 24 h.

Step 2: In the second step, TyroFill scaffolds were prepared by immersing E1001(1k) scaffolds in 1 M CaCl_2_ solution. To ensure that the solution fills the entire pore volume, the immersed scaffolds were first exposed to a vacuum of up to 30 inHg for 1 min, followed by rapid release to atmospheric pressure. This was repeated 5 times. Next, the scaffolds were immersed in 1 M K_2_HPO_4_ solution, and the vacuum treatment was repeated 5 times as before. The scaffolds were alternated in CaCl_2_ and K_2_HPO_4_ solutions for 3 cycles. This resulted in the formation of a coating of DCPD throughout the pore volume of the scaffold. The resulting TyroFill (E1001(1k)-DCPD) scaffolds were dried, placed in ETO sterilization pouches, and sterilized using an ethylene oxide (EtO) sterilizer (AN74i, Anderson Products, Haw River, NC, USA). After sterilization, the sealed scaffolds were stored at −20 °C until use.

### 2.2. Cell Seeding

Before cell seeding, TyroFill scaffolds were pretreated in mesenchymal cell osteogenic media (OM) (DMEM/F12, 10% FBS, 1% GlutaMAX, 100 nM dexamethasone, 10 mM beta-glycerolphosphate, 50 µg/mL ascorbic acid, and 1% penicillin/ streptomycin/ amphotericin (PSA)) for one week, to further develop a calcium coating [28].

hDPCs were harvested and characterized as previously described by us [24]. Briefly, teeth were extracted by trained clinicians at the Tufts University School of Dental Medicine (TUSDM) using Tufts University IRB-approved protocols. The dental pulp was then harvested from the extracted teeth, minced into small pieces, and digested using 0.4 mg/mL collagenase type I (Sigma-Aldrich, St. Louis, MO, USA) and 0.2 mg/mL dispase (Boehringer Mannheim, Indianapolis, IN, USA) to generate single-cell suspensions. hDPSCs were in vitro cultured and expanded in 5% CO_2_ at 37 °C in dental mesenchymal cell medium with DMEM/F12, 10% FBS, 1% GlutaMAX, 25 µg/mL ascorbic acid, and 1% PSA, and then cryopreserved until use. The multipotent (osteogenic, chondrogenic, adipogenic, and neurogenic) differentiation potential of each hDPSC cell line was confirmed prior to use. HUVECs were expanded in vascular basal media (PCS100030, ATCC) with VEGF growth kit (PCS100041, ATCC) in 5% CO_2_ at 37 °C and cryopreserved at passage three.

Both types of cryopreserved cells were thawed and expanded in vitro immediately prior to implant fabrication. Equal numbers (1:1) of hDPSCs and Human Umbilical Vein Endothelial Cells (HUVECs, ATCC, PSC100010, Manassas, VA, USA) were seeded dynamically onto TyroFill scaffolds for a final density of 0.25 × 10^5^ cells/mm^3^. HUVECS were used to facilitate the vascularization of the implanted construct. Cell-seeded and unseeded acellular scaffolds were in vitro cultured in 1:1 DPSC:HUVEC medium with osteogenic supplements listed above for one week prior to in vivo implantation to ensure sufficient time for cell attachment and proliferation and to initiate differentiation prior to implantation. Titanium (Ti) implants (SLActive^®^ 8, Straumann, Andover, MA, USA) were screwed into each TyroFill scaffold and cultured for an additional three days prior to implantation. Since the Ti implants used in this study had an expired sterilization date, all implants were autoclaved prior to their use.

### 2.3. Rabbit Mandible Defect Repair Model

**Figure 1:**
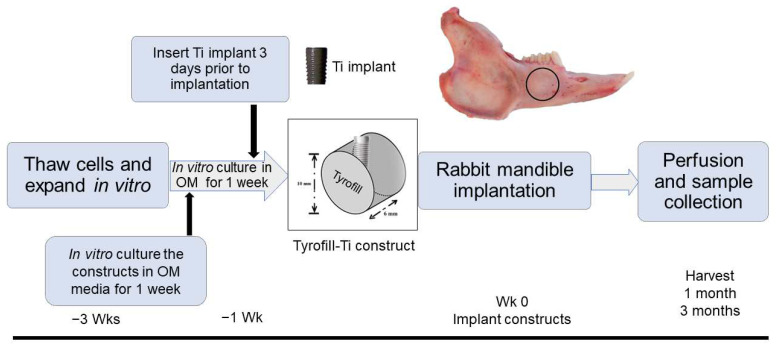
In vivo TyroFill-Ti implant study design. Detailed schematic of the implant study. The black circle indicates the location of the rabbit mandible defect site.

All animal experiments were conducted under the guidance and approval of the Institutional Animal Care and Use Committee (IACUC) of Tufts University. The rabbit mandible defect repair model used in this study was performed on New Zealand White Rabbits (>3.5 kg) [20]. For each time point (1 and 3 months implantation), experimental samples consisted of TyroFill scaffolds containing implants that were cell-seeded (*n* = 3) or acellular (*n* = 2), one implant per rabbit, 5 rabbits per time point (Figure 1). To achieve a 95% confidence level and 10% standard deviation, we used 5 animals per time point (*n* = 2). Briefly, fully anesthetized rabbits were placed in a dorsal position, and a midline incision was made under the chin, followed by the dissection of the fascia and muscle to expose the left side mandibular bone. A full-thickness mandibular bone defect was made through the roots of the second molar using a 10 mm trephine bur under copious sterile saline irrigation. Buccal cortex bone, exposed tooth roots, and lingual cortex bone were sequentially removed with a periosteal elevator to create a full-thickness defect, and the defect site was thoroughly irrigated with sterile saline to remove any remaining bone and tooth fragments. Next, a cell-seeded or acellular TyroFill with a dental implant was placed into the defect, and 4–0 Vicryl was used to close the overlying muscle and skin layers. Heart rate, oxygen saturation, carbon dioxide, respiratory rate, and body temperature were monitored carefully throughout the procedure. A soft, critical-care diet was provided to experimental rabbits for 2 weeks post-operation. After 1 or 3 months of implantation, implanted and contralateral unoperated control jaws were harvested using formalin perfusion to ensure sufficient fixation of implant tissues. The harvested mandibles were then re-fixed in 4% formalin for 3–5 days with rocking at room temperature, hemisected using a band saw, analyzed via Micro-CT, demineralized, and processed for histological and immunohistochemical analyses.

### 2.4. Evaluation of Bioengineered Mandibular Bone Implants

Harvested hemi-mandibles (*n* = 10 per time point, 5 control unoperated and 5 implanted) were scanned using a microcomputed tomography (µCT) imaging system (Skyscan 1176, Bruker MicroCT, Billerica, MA, USA). Scans were performed on all harvested implants using the set parameters of 100 kV, 100 A, Al-Cu filter, 0.3 rotation step over 180°, and pixel size 9 μm, together with two BMD phantoms with BMD values of 0.25 and 0.75 g/cm^−3^. µCT data were then reconstructed to rebuild acquisition datasets using NRecon 2.0 software (Bruker MicroCT). The region of interest (ROI), defined as a full-thickness (6 mm), 10 mm diameter circle that matched the defect area, was further selected and evaluated for new bone regeneration using Avizo 9.1 (Version 1.6.9.15, ThermoFisher Scientific, Materials and Structural Analysis Division, Hillsboro, OR, USA) and CTAn 1.18 (Bruker MicroCT) software. A full-thickness 10 mm diameter, 6 mm wide region on the unoperated control right side mandible was similarly analyzed (Supplementary Figure S1, Supplementary Materials). Harvested hemi-mandibles were then decalcified in (1:1) 45% formic acid:20% sodium citrate solution for one month. The Ti dental implants were carefully removed for subsequent SEM analyses, and the remaining bone implants were prepared for paraffin embedding and sectioning and histological/IF analyses. Paraffin sections were analyzed using Hematoxylin and Eosin (H&E) and Masson’s Trichrome staining. Immunofluorescent (IF) staining was performed using primary antibodies for the odontoblast differentiation marker dentin sialophosphoprotein (DSPP, abx176139, Abbexa Ltd., Cambridge, UK) and α-smooth muscle actin (SMA, Ab21027, Abcam, Cambridge, UK) for blood vessel formation, and anti-human MHC class I + HLA A + HLA B antibody (Ab134189, Abcam) to detect any human DPSCs/HUVECs in the implants. To evaluate hDPSCs and HUVEC distribution throughout TyroFill scaffolds prior to implantation, replicate (2) cell-seeded constructs were embedded in OCT, cryosectioned, and subjected to histological and IF analyses. IF staining was performed on replicate (5) sections that spanned each scaffold using the primary antibodies for the mesenchymal cell marker Vimentin (VM, sc-6260, Santa Cruz Biotechnology, Dallas, TX, USA), the endothelial cell marker Factor VIII (ab61910, Abcam), and appropriate secondary antibodies. Replicate (3) 40 × images were taken on each section by an M2-Bio Zeiss fluorescent microscope (Zeiss, Jena, Germany). Positive cells were identified and quantified via Image J software 1.53 (National Institutes of Health, Bethesda, MD, USA).

### 2.5. Surface Characterization of the Titanium Dental Implants

The surface morphology and elemental composition of un-implanted control and implanted Ti dental implants (*n* = 10) were analyzed via Scanning Electron Microscope (SEM) and Energy-Dispersive X-ray (EDAX), respectively. Ti dental implants were dehydrated in graded ethanol series and HMDS (Hexamethyldisilazane), sputter coated by gold/palladium, and analyzed by SEM (Hitachi S-4800, Tokyo, Japan) at the Northeastern University EM Facility (Boston, MA, USA). Qualitative chemical composition at three different sites on each Ti implant was assessed by EDAX (Oxford Instruments EDX detector). Two non-implanted starting material Ti dental implants were processed and analyzed as controls.

## 3. Results

### 3.1. Construction and In Vitro Culture of 3D Bone–Tooth Constructs

**Figure 2:**
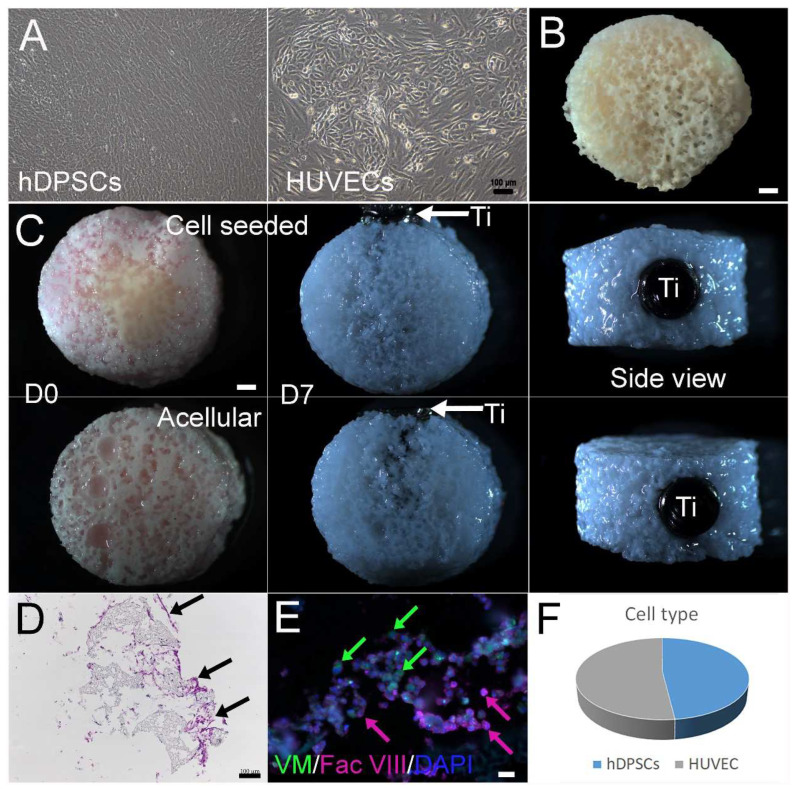
Characteristics of TyroFill-Ti constructs: hDPSCs) (**A**) and HUVECs (**B**) showed typical morphologies prior to cell seeding. (**C**) TyroFill-Ti constructs after 1-week in vitro culture in OM, with cells (upper panel) or without cells (lower panel). D0 indicates scaffolds immediately after cell seeding, and D7 indicates scaffolds immediately prior to implantation. (**D**) Histological analysis showed abundant cell distribution throughout the TyroFill construct before implantation. Black arrows indicate some cell clusters. (**E**) Double IF staining revealed both hDPSCs (green) and HUVECs (pink) in TyroFill implant prior to implantation. Color arrows point out some of the positive cells accordingly. (**F**) Statistical analysis showed that hDPSCs and HUVEC retained a ~1:1 ratio prior to implantation. Abbreviations: Ti, Titinium implant; VM, Vimentin; Fac VIII, factor 8. Scale bars: (**B**,**C**) 1 mm; (**A**,**D**) 100 µm; (**E**) 20 µm.

In vitro, expanded hDPSCs and HUVECs exhibited a healthy appearance prior to seeding onto scaffolds (Figure 2A). TyroFill scaffold fabrication was performed as previously published [28]. No obvious changes were observed in cell-seeded or acellular TyroFill scaffolds after one week in vitro culture in osteogenic media (Figure 2B). The inserted dental implant remained stable prior to implantation (Figure 2C). Histological analyses of cryosectioned cell-seeded constructs prior to implantation stained with H&E revealed good cell attachment and morphology throughout the constructs (Figure 2D). Immunostaining of mesenchymal cell marker Vimentin and endothelial cell marker Factor VIII revealed that hDPCs and HUVECs remained at an approximate 1:1 ratio after 1 week of culture in osteogenic media (Figure 2E,F).

### 3.2. Post-Surgical Analyses

**Figure 3:**
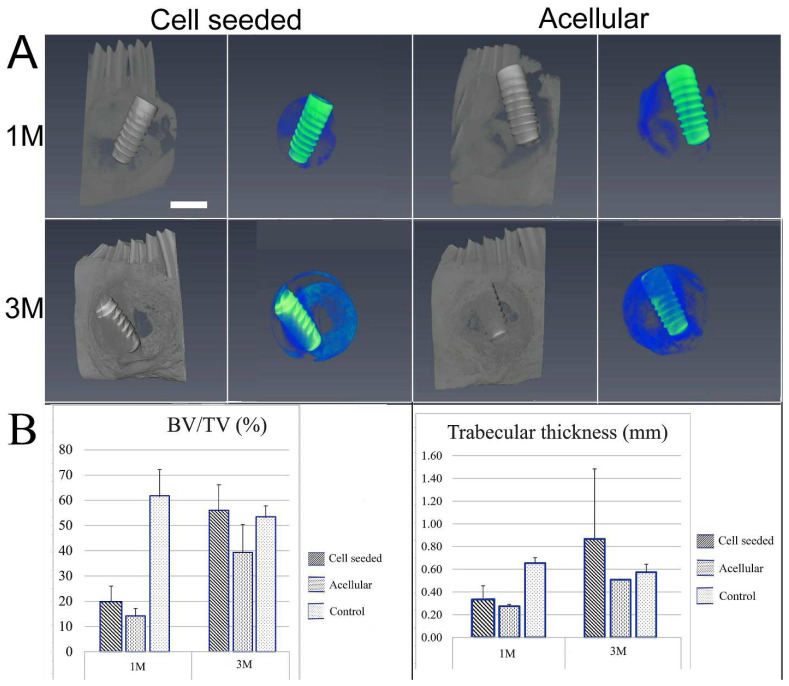
Microcomputed tomography (µCT) analyses of harvested implants: (**A**) Representative 3D µCT images of harvested 1-month (upper panel) and 3-month (lower panel) implants. A full-thickness 10 mm diameter cylindrical area (blue color) that matched the defect area was selected, and the Ti implant was excluded based on its distinctive density (green). Increased amounts of radiopaque calcified tissue were observed in 3-month as compared to 1-month implanted constructs. hDPSCs/HUVEC-seeded TyroFill constructs exhibited more homogeneous mineralized tissue formation throughout the implants as compared to acellular constructs. (**B**) Quantification of new bone volume/tissue volume (BV/TV) and trabecular thickness within the constructs at the implant site. Cell-seeded constructs at later time points showed greater and more mature bone formation. Error bars indicate the standard deviation among samples. Scale bars: (**A**) 2 mm.

All rabbits showed excellent recovery and healing after surgery, and no weight loss or other adverse reactions were observed. After 1 and 3 months, no noticeable changes in the dentition or jawbone were observed in any of the implanted jaws as compared to the contralateral unoperated control mandible. Three-dimensional (3D) µCT analyses of the harvested jaws with implants showed an easily identifiable radiolucent circular defect site and highly radiopaque dental implant in all harvested mandibles at 1 and 3 months (Figure 3A). Radiopaque areas at the implant site indicated newly formed bioengineered mineralized tissue. Comparatively, more mineralized tissue formation was observed at 3 months as compared to 1 month post-implantation. hDPSC-HUVEC cell-seeded implants exhibited more uniform calcified tissue formation throughout the entire implant site as compared to implanted acellular constructs (Figure 3A). New hard tissue formation was quantified for bone density and bone volume/tissue volume (BV/TV) measurements within the selected defect site area using µCT image analyses (Figure 3B). Trabecular thickness was quantified to evaluate the maturity of newly formed bone (Figure 3). Both measurements showed that cell-seeded constructs exhibited increased bone volume and maturity over time as compared to acellular construct implants, although no significant difference was observed due to the limited number of implants. Comparatively, TyroFill constructs showed more robust new bone formation as compared to previously characterized E1001(1K)-bTCP scaffolds implanted using similar conditions [25] (Supplementary Table S1).

### 3.3. Histological Analyses of Bioengineered Constructs

**Figure 4:**
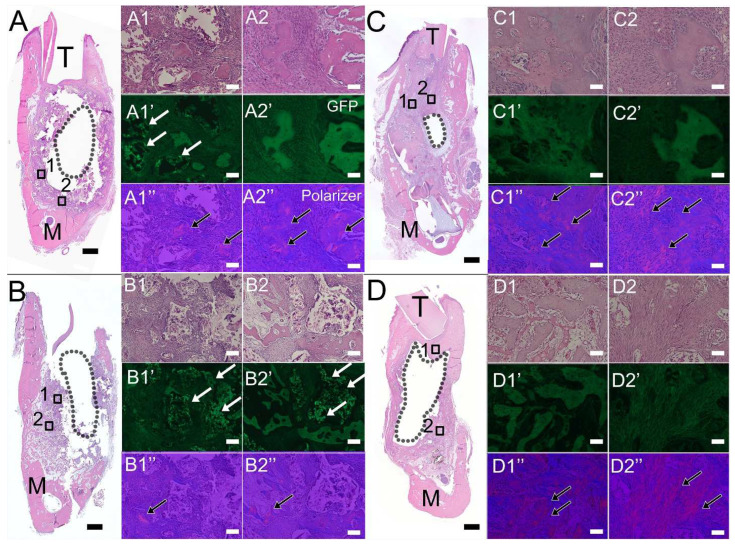
Histological analysis of harvested TyroFill implants: (**A**) H&E stained 1-month hDPSCs/HUVEC-seeded and (**B**) acellular TyroFill constructs, and (**C**) 3-month hDPSCs/HUVEC-seeded and (**D**) acellular TyroFill constructs. Panels 1 and 2 show high-magnification images of boxed areas in (**A**–**D**). The void left by the removed Ti implant is outlined as shown. After 1 month of implantation, cell-seeded constructs (**A1**,**A2**) showed more obvious new bone formation than the acellular constructs (**B1**,**B2**). After 3 months of implantation, cell-seeded constructs showed new bone formation throughout (**C1**,**C2**), while no obvious bone formation was evident in acellular constructs (**D1**,**D2**). **A1’**–**D1’** and **A2’–D2’** show the remaining scaffold using GFP filter. White arrows indicate undegraded TyroFill in green. Corresponding polarized light images (**A1”**–**D1”** and **A2”**–**D2”**) confirm new bone formation. Black arrows indicate areas of mature new bone formation. Abbreviations: M, mandible; T, tooth. Scale bar = 1 mm (**A**–**D**), 50 µm (all other panels).

H&E staining of coronally sectioned harvested constructs was used to assess bioengineered bone formation at the defect site. Histological analyses showed that most TyroFill scaffold pores were filled with well-organized soft tissues in 1-month cell-seeded samples, while 1-month acellular samples exhibited reduced soft tissue density and volume. Bioengineered bone fragments similar to that of natural bone were detected in cell-seeded 1-month implants (Figure 4A). After 3 months of implantation, cell-seeded TyroFill constructs showed robust bone formation throughout the entire defect area (Figure 4C). Although new bone formation was observed around the periphery of the implant area, no obvious bone formation was present near the center of acellular constructs (Figure 4D). E1001(1k), the polymer used in the manufacture of the TyroFill scaffold, appeared green in 1-month implants when viewed using a fluorescent GFP filter, particularly in acellular implants (Figure 4A,B GFP panel, indicated by arrows). No noticeable polymer was observed in TyroFill constructs after 3 months of implantation (Figure 4C,D).

**Figure 5:**
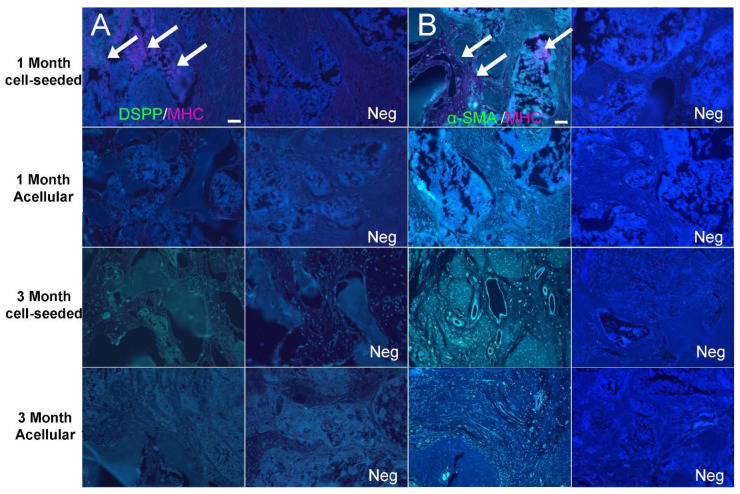
Immunofluorescent analyses of bone differentiation marker expression in bioengineered bone TyroFill constructs. Representative images taken of sections obtained from the center of harvested implants. DSPP (green) was detected in all harvested implants, with strongest expression in 3-month hDPSCs/HUVEC-seeded implants (**A**). Strong α-SMA expression (green) indicated the presence of blood vessel formation in the center of the implants, especially in 3-month harvested cell-seeded implants (**B**). MHC-positive human DPSCs/HUVECs (red) were only detected in 1-month hDPSCs/HUVEC-seeded implants. White arrows indicate human (MHC+) cells expressing DSPP or α-SMA. Scale bar = 50 µm.

Immunofluorescent staining performed using bone/dentin markers also indicated robust bioengineered alveolar bone formation, particularly in DPSC/HUVEC-seeded TyroFill constructs. Positive DSPP expression was observed in all harvested implants at 1 and 3 months, with stronger expression observed in cell-seeded constructs, especially after 3 months of implantation (Figure 5A, green). Strong α-SMA expression (green) indicated blood vessel formation throughout the implants, especially in 3-month cell-seeded implants. MHC-positive human DPSCs/HUVECs (red) were only detected in 1-month hDPSCs/HUVEC-seeded TyroFill constructs (Figure 5).

### 3.4. Characterization of Harvested Dental Implant Surfaces

**Figure 6:**
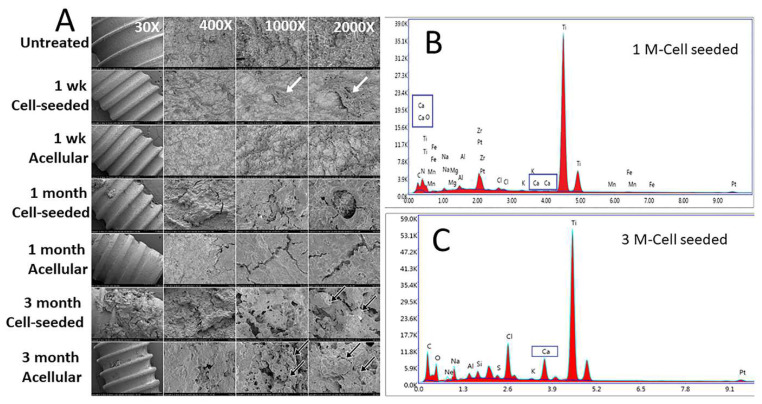
Analyses of Ti implant Surfaces. SEM (**A**) and EDAX (**B**,**C**) analysis of Ti dental implants removed from harvested TyroFill constructs indicated calcified matrix deposition on the implant surface. White arrows indicate areas containing cells. Black arrows indicate calcified nodule formation. Representative EDAX spectrum of the surface of Ti implants harvested from cell-seeded TyroFill constructs after 1 month (**B**) and 3 months (**C**) implantation. The elemental spectral peaks include significant amounts of titanium and calcium, as indicated.

SEM analyses of un-implanted Straumann Ti dental implants showed an irregular honeycomb structure (Figure 6A) similar to that of previously published reports [29]. No significant difference was observed on the surface untreated and 1-week in vitro cultured dental implants (Figure 6A). After in vivo implantation at 1 and 3 months, the surface of all acellular and cell-seeded Ti implants exhibited deposition of highly organized extracellular matrix (ECM). Implants removed from the cell-seeded constructs exhibited clearly identifiable cells on the surface (2000×) (Figure 6A). Relatively increased calcified nodule formation was observed on the surface of implants retrieved from 3-month cell-seeded constructs as compared to 1-month implanted constructs. As expected, Energy-Dispersive X-ray (EDAX) analyses revealed the presence of titanium on the surface of all Straumann Ti dental implants (Figure 6B). EDAX analysis also confirmed surface deposition of bone matrix containing higher levels of Ca in cell-seeded constructs (Figure 6B). No significant difference was found between cell-seeded and acellular samples, likely due to insufficient sample numbers (Supplementary Table S2).

## 4. Discussion

Successful therapies to regenerate bone in large, critical-sized CMF defects require bone grafts that exhibit high biocompatibility, sufficient mechanical properties to support CMF structure and masticatory function during the bone healing process, and the ability to be easily handled and accurately shaped to precisely fit the uniquely complex anatomies of craniomaxillofacial bones [30,31]. Ideally, these scaffolding materials should also be biodegradable and eventually be replaced by robust and vital newly formed bone. Autogenous bone is considered the current gold standard, and autogenous cryogenically preserved autogenous extracted teeth have also been proposed as another source of natural mineralized tissue for tissue regeneration [32].

Although the mechanisms regulating carbonate hydroxyapatite (CAP)-induced bone formation are still incompletely understood, the superior osteoinductive properties of CAP make it one of the best scaffolds for bone regeneration [33]. For example, one study used CAP granules combined with stem cells from human exfoliated teeth (SHEDs), transplanted to the defect using an atelocollagen sponge scaffold, to treat calvarial defects in immunocompromised 6-week-old male immunodeficient mice. Analyses of these harvested implants clearly showed that the SHEDs + CAP transplantation group exhibited significantly higher bone regeneration as compared to the CAP alone and SHEDs alone groups [34]. Ideally, the most promising scaffolds would consist of biomimetic organic scaffolds coated with CaP to mimic the organic–inorganic composition of native bone [35]. Our previously published results showed that E1001(1k) derived porous scaffolds supported robust bone regeneration in a rabbit critical-sized calvarial defect and in an ovine long bone defect repair model [20,21,22]. Our previously published results also demonstrated that E1001(1K)-βTCP scaffolds are very effective for mandibular jaw regeneration in a critical-sized rabbit mandible defect model [25]. Based on these promising results and the fact that a new E1001(1k) formulation, TyroFill, also effectively supports in vitro cultured DPSC proliferation and differentiation [28], the objective of this study was to test whether hDPSC/HUVEC-seeded TyroFill scaffolds were effective in repairing alveolar bone in an in vivo rabbit mandibular defect repair model. The TyroFill scaffolds used in this study exhibited bimodal, interconnected macro- and microporous structures with >90% final porosity that mimicked the pore size range and architecture of trabecular bone [28].

Our results showed mineralized tissue regeneration within and on the surface of both hDPSC/HUVEC-seeded and acellular TyroFill scaffolds, in accordance with the highly porous and osteoconductive nature of these scaffolds. The presence of new bone formation within and on the surface of acellular Tyrofill scaffold implants indicates the ability of TyroFill scaffolds to recruit host cell participation in mineralized tissue regeneration. µCT and histological analyses showed that rabbit mandibles implanted with hDPSC/HUVEC-seeded TyroFill scaffolds exhibited a unique pattern of mineralized tissue formation as compared to acellular scaffolds. Fewer but larger areas of calcified tissue formed largely in the periphery and not the center of acellular scaffold implants, while smaller areas of homogeneous and evenly distributed new bone formed throughout hDPSC/HUVEC-seeded constructs. Furthermore, the expression of DSPP, a dentin-specific matrix protein also expressed in naturally formed alveolar bone, was only observed in bioengineered bone derived from hDPSC/HUVEC-seeded TyroFill constructs and not in acellular constructs. Together, these results suggest that TyroFill scaffolds exhibit the ability to support hDPSC differentiation, vascularized tissue formation, and the formation of mineralized tissue resembling that of natural jawbone. We further demonstrated that MHC expressing hDPSC/HUVECs were detectable in cell-seeded TyroFill implants harvested at 1 month but not at 3 months. These results are consistent with numerous reports showing that implanted human cells contribute to long-term tissue regeneration but do not maintain long-term residence in the implants [36].

In natural bone formation and remodeling, osteogenesis and angiogenesis are tightly coupled processes [37,38]. Blood vessels not only carry oxygen and nutrients to developing bone but also play an active role in mediating interactions between osteoblasts, osteocytes, osteoclasts, and endothelial cells [39,40]. A unique property of E1001(1k) derived scaffolds is their highly organized micro-architecture consisting of a highly interconnected porosity that facilitates efficient cell infiltration through macropores (200–400 μm) and efficient delivery of nutrients through micropores (<20 μm). For the study described here, 10 mm diameter × 6 mm high cylindrical TyroFill scaffolds were seeded with both HUVECs and hDPSCs to facilitate angiogenesis and alveolar bone formation, respectively. In fact, our results showed significant blood vessel formation throughout the cell-seeded implanted constructs, especially after 3 months of implantation.

With respect to facilitating the osseointegration of Ti implants, a porous scaffold could increase long-term mechanical stability by facilitating bone growth into the highly porous scaffold and around the Ti implant [41]. It was previously shown that a pore size range of 100–600 μm could promote efficient osseointegration [42,43]. The TyroFill scaffold with Ti implant used in this study exhibited similar pore size, which efficiently promoted calcified tissue formation throughout the TyroFill scaffold and around the Ti implant, particularly in cell-seeded constructs.

In summary, the studies described here demonstrate the potential for hDPSCs-HUVEC-seeded TyroFill constructs as a potential new and improved therapy to efficiently repair CMF defects. TyroFill constructs exhibited better bone-forming capability as compared to previously used hDPSC-seeded E1001(1k)/β-TCP scaffolds, indicating the importance of the DCDP coating for bone regeneration. The observed, robust new bone formation within TyroFill scaffolds and on the surface of Ti implants, especially in cell-seeded constructs, indicates the potential utility of TyroFill-Ti scaffolds as a potentially new and more effective therapy for coordinated CMF bone and tooth regeneration to improve patient outcomes and reduce surgical costs.

## Data Availability

All published data are available upon request.

## Supplementary Materials

The following supporting information can be downloaded at https://www.mdpi.com/article/10.3390/bioengineering10111277/s1, Figure S1: Microcomputed tomography (µCT) and histology of unoperated control mandibles. Table S1: Microcomputed tomography (µCT) data of harvested implants. Table S2: EDAX analysis of Ti Dental Implant Surfaces. Video S1: µCT analysis of a cell-seeded construct after 1 month implantation. Video S2: µCT analysis of an acellular construct after 1 month implantation. Video S3: µCT analysis of an unoperated control mandible at 1 month. Video S4: µCT analysis of a cell-seeded construct after 3 months implantation. Video S5: µCT analysis of an acellular construct after 3 months implantation. Video S6: µCT CT analysis of an unoperated control mandible at 3 months.
