# Supplementary material for: TyroFill–Titanium Implant Constructs for the Coordinated Repair of Rabbit Mandible and Tooth Defects
Source: Bioengineering (Basel). 2023 Nov 2;10(11):1277. doi: 10.3390/bioengineering10111277 (PMC10668976; doi:10.3390/bioengineering10111277)
Supplement: Supplementary file 1 [file bioengineering-10-01277-s001.zip › Supplemental Data - Bioengineering 2023 110123.pdf]

bioengineering

*Supplementary Materials*

# TyroFill–Titanium Implant Constructs for the Coordinated Repair of Rabbit Mandible and Tooth Defects

**Weibo Zhang [1], Joachim Kohn [2] and Pamela C. Yelick [1,*]**

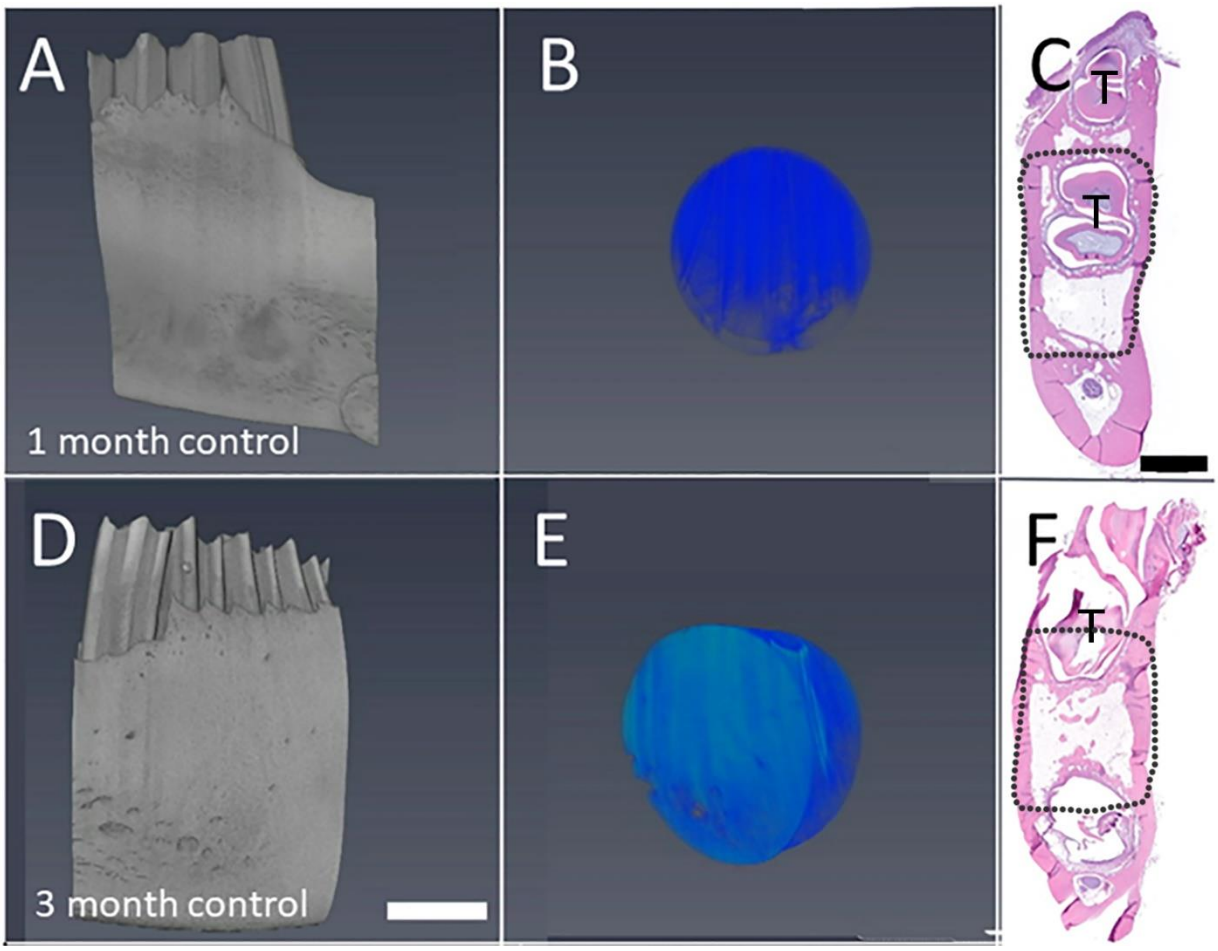


**Figure S1.** Microcomputed tomography (μCT) and histology of unoperated control mandibles. (**A**,**B**) 1 month and (**D**,**E**) 3 months control mandibles. (**A**,**D**) 3D reconstructed μCT data. (**B**,**E**) A selected 10 mm x 6 mm region of interest. **H**,**E** stained sectioned 1 month (**C**) and 3 months (**F**) control mandibles. Dotted lines indicate the approximate position of implant placement. Abbreviations: T, Tooth. Scale bars = 2mm.

**Table S1:** Microcomputed tomography (μCT) data of harvested implants. TryoFill constructs showed improved new bone formation as compared to previously used E1001(1K)-bTCP constructs. No significant differences were observed due to the limited number of implants placed in this study.

| **TryoFill** | | **Bone Density** | **BV/TV (%)** | **Trabecular Thickness (mm)** |
|---|---|---|---|---|
| 1M | Cell seeded | 0.65 ± 0.02 | 19.65 ± 5.87 | 0.33 ± 0.13 |
| | Acellular | 0.70 ± 0.11 | 15.35 ± 3.81 | 0.26 ± 0.00 |
| | Control | 0.67 ± 0.13 | 61.12 ± 11.02 | 0.65 ± 0.05 |
| 3M | Cell seeded | 0.38 ± 0.08 | 56.05 ± 10.13 | 0.87 ± 0.52 |
| | Acellular | 0.56 ± 0.01 | 39.42 ± 10.98 | 0.51 ± 0.00 |
| | Control | 0.62 ± 0.08 | 53.50 ± 4.22 | 0.58 ± 0.07 |

| **E1001(1K)-bTCP** | | **Bone Density** | **BV/TV (%)** | **Trabecular Thickness (mm)** |
|---|---|---|---|---|
| 1M | Cell seeded | 0.54 ± 0.06 | 5.79 ± 2.47 | 0.07 ± 0.04 |
| | Acellular | 0.46 ± 0.12 | 13.05 ± 6.8 | 0.11 ± 0.01 |
| | Control | 0.47 ± 0.24 | 49.67 ± 0.00 | 0.264 ± 0.00 |
| 3M | Cell seeded | 0.52 ± 0.06 | 19.45 ± 10.57 | 0.19 ± 0.11 |
| | Acellular | 0.4 ± 0.10 | 18.51 ± 0.00 | 0.19 ± 0.00 |
| | Control | 0.42 ± 0.21 | 42.94 ± 0.00 | 0.43 ± 0.00 |

**Table S2:** EDAX analysis of Ti Dental Implant Surfaces. Calcium was not detected on the surface of unimplanted control or 1-week *in vitro* cultured acellular Ti dental implants. Calcium was detected on the surface of Ti dental implants removed from 1 month and 3 month implanted constructs, although no obvious trend was observed likely due to the limited number of samples used in this study.

| $Ca^{++}$ | % Weight | |
|---|---|---|
| | Average | SD |
| Unoperated Control | 0.00% | 0.000 |
| 1W Cell seeded | 0.17% | 0.002 |
| 1W Acellular | 0.00% | 0.000 |
| 1M Cell seeded | 1.04% | 0.013 |
| 1M Acellular | 0.49% | 0.005 |
| 3M Cell seeded | 0.13% | 0.001 |
| 3M Acellular | 0.62% | 0.007 |

**Supplemental Videos 1–6:**

**Video S1**. µCT analysis of a cell-seeded construct after 1 month implantation. Scattered radio-opaque areas indicate calcified tissue formation throughout the implant site.

**Video S2**. µCT analysis of an acellular construct after 1 month implantation. Very few radio-opaque areas of calcification were observed throughout the implant site.

**Video S3**. µCT analysis of an unoperated control mandible at 1 month.

**Video S4**. µCT analysis of a cell-seeded construct after 3 months implantation. Highly calcified mineralized tissue nodules indicative of new bone formation were observed throughout the implanted site.

**Video S5**. µCT analysis of an acellular construct after 3 months implantation. Newly formed bone can be observed throughout the implant, but less than seen in cell-seeded constructs.

**Video S6**. µCT CT analysis of an unoperated control mandible at 3 months.
